# Early language screening at 30 months of age in Swedish child health services—a qualitative study of nurses' perspectives

**DOI:** 10.3389/fped.2025.1570793

**Published:** 2025-06-25

**Authors:** AnnaKarin Larsson, Amanda Häll, Emma Granér, Emilia Carlsson

**Affiliations:** ^1^ Speech and Language Pathology Unit, Department of Health and Rehabilitation, Institute of Neuroscience and Physiology, Sahlgrenska Academy, University of Gothenburg, Gothenburg, Sweden; ^2^ Child Health Unit, Regionhälsan, Region Västra Götaland, Gothenburg, Sweden

**Keywords:** nursing, health visits, child health, language screening, language disorders, child-health services, qualitative content analysis, multilingualism

## Abstract

**Aim:**

To understand how nurses within the child-health services perceive the early language screening administered at 30 months of age.

**Methods:**

A qualitative study was conducted involving individual interviews with 15 nurses working in the child-health services of two districts in western Sweden. The interview data were analysed through content analysis.

**Results:**

The qualitative analysis yielded two main categories: (1) *Experience and flexibility facilitate use of the screening method* and (2) *External factors influence administration as well as the assessment and analysis of screening results*. Regarding the first main category, the nurses considered that the screening method often worked well, but their confidence in using it was influenced by the length of their working experience. The second main category highlights external factors influencing the nurses' administration of screenings and their analysis and assessment of screening results, such as the child's abilities and overall development, language barriers, parental expectations and waiting times in healthcare. The two main categories can be broken down into seven sub-categories.

**Conclusions:**

Our findings indicate that nurses' experiences with interpreting screening results vary depending on their professional background and on the children's abilities, with particular challenges arising in the case of immigrant children.

## 1 Introduction

Although there is no international recommendation to use language screening in primary care, the Swedish child-health services (CHS) have used different speech-and-language screening tools since the early 1970s (1). The aim is to detect delayed language development and children in risk of having a language disorder. Delayed language development and language disorder are important neuro-developmental symptoms and conditions that may strongly affect a child's future development. For instance, children with language disorder are at great risk of academic failure and mental illness if their language difficulties are not addressed (2). In addition, language disorder is much more prevalent than other neuro-developmental conditions, affecting roughly 9% of children (3). For these reasons, language disorder has been identified as a major public-health concern (4).

The use of language screening in CHS is not without controversy, and most countries discourage its implementation in primary care settings (5, 6). While the notion of early identification of children in need of language development support is well-founded, it is important to recognize that language and speech development often vary significantly among children during the pre-school years (7). This makes it difficult to determine whether the right children are being accurately identified at an early stage. Moreover, it is crucial that children and their families are offered timely and appropriate early intervention once specific needs have been identified—something that most regions in Sweden currently struggle to provide (8).

Since the early 1990s, two methods have been used in Sweden, one administered at the age of 30 months (1) and the other at 36 months (9). Today, all Swedish child-health centres (CHC), to which most children come for regular health visits, use one of them (10). They have been evaluated in a Swedish context and found to have acceptable to good validity in terms of specificity and sensitivity (1, 11). The present study focuses on the language screening administered at 30 months of age. This method is used by approximately half of all CHCs in Sweden today. In some regions it has been used since 1991 (1) and the method has been modified and adapted into Swedish conditions based on a method from the UK (1, 12, 13). It includes a short parental questionnaire and observations by a child-health (CH) nurse (1, 10). The observations are made in accordance with a protocol where the nurse uses toys to engage with the child. The main focus is to assess whether the child understands simple instructions given by the nurse and whether the child uses two-word sentences. Further, the protocol includes assessing if the child talks spontaneously, can name simple objects, can imitate, understands short sentences including the prepositions in/on, and if the child responds and participates adequately during the assessment (1). In some regions of Sweden, nurses receive training and support in the language screening method they use. The training is provided by a speech-language pathologist within CHS. However, in most regions, this is lacking, and the nurses have to rely solely on the screening manual.

Apart from investigating the validity of a screening method, it is important to investigate how it is administered, to ensure compliance and fidelity. A previous study found that Swedish CH nurses experienced difficulties using language screening with bilingual children, causing them to deviate from the protocol and to delay referrals of children having failed the screening (14). Another more recent study found that children having experienced adverse life events posed particular difficulties for CH nurses; the authors in fact questioned the validity of the screening method (15). However, there are still very few such studies. More research is needed to better evaluate the usefulness of early language screening. The present study aimed to help fill a gap in our knowledge about early language screening within the CHS by enhancing our understanding of how child-health nurses who administer such screenings perceive them.

## 2 Method

### 2.1 Participants

Child-health (CH) nurses in two geographical areas within the Västra Götaland region were recruited through purposive sampling. Demographically, the two areas include both rural and urban areas. Managers of child-health centres were contacted to reach child-health nurses at those centres. A total of 18 nurses were reached and 15 of them gave their written informed consent to be part of the study. See Table 1 for data on their experience working in child health and with language screening, collected through interviews.

**Table 1:** Participating child-health nurses (*n* = 15).

Experience	Range
Time spent working in the child-health services	3–25 years (*Md* = 7)
Experience using the screening method	3–14 years (*Md* = 6.5)
Typical frequency of screenings of 2.5-year-olds	Once every two weeks–daily

### 2.2 Data collection and analysis

Semi-structured individual interviews with nurses were performed online by two of the authors in January and February 2022, based on an interview guide created by the authors. The interviews lasted for 30–60 min, were audio-recorded and transcribed verbatim. Examples of questions in the interview guide were: *What are your perceptions of using language screening tools with 2.5-year-old children? How do you perceive the challenges or facilitators involved in interpreting language screening results? Have you observed any variations or challenges in administering the language screening across different children or contexts?*

To analyse the data, content analysis with an inductive approach was used (16). All transcriptions were first read through, and then meaning units were identified. The meaning units were structured and analysed using the NVivo software, coded and then sorted into categories and sub-categories based on similarities and differences. The aim was to find categories that were exhaustive and mutually exclusive. To strengthen credibility, all authors were involved in the discussion of categories and sub-categories (17). For an example of the analysis, see Table 2.

**Table 2:** Example from the analytic process.

Quote	Meaning unit	Code	Subcategory	Category
“*It has also become easier over the years, as I've gained more experience—you can more easily see which children are lagging behind in their language development compared to those who are developing in line with the expected language level at the 2.5-year check-up*”	It has become easier over the years, with more experience, to identify which children are behind in their language development compared to those who are in line with typical development	Easier to decide what to do with greater experience	Work experience gives confidence in administering the screening	Experience and flexibility facilitate use of the screening method

### 2.3 Ethical considerations

The ethical principles laid down in the World Medical Association's Declaration of Helsinki were followed. All participants gave their written informed consent, and the study was approved by the Swedish Ethical Review Authority (Ref. No. 2021-05763-01).

## 4 Results

The analysis yielded two main categories and seven sub-categories as described below and shown in Table 3. All quotations have been translated from Swedish to English by the authors.

**Table 3:** Categories and sub-categories that emerged from content analysis.

Category	Subcategory
Experience and flexibility facilitate use of the screening method	Work experience gives confidence in administering the screening
	A need for complementary approaches to capture the overall picture
	A family-centred approach gives the best results
External factors influence administration as well as the assessment and analysis of screening results	The child's abilities affect administration
	Language barriers affect administration and assessment
	Parents' perceptions have an impact
	Decisions are influenced by waiting times in healthcare.

### 4.1 Experience and flexibility facilitate use of the screening method

Experience helped the nurses interpret and analyse assessment results, which was sometimes a difficult task. To be able to serve the children's and their families best interests, the nurses needed flexibility and had to think outside the box.

#### 4.1.1 Work experience gives confidence in administering the screening

Experience from working in child health helped the nurses to feel secure and confident. Longer work experience also made it easier to interpret whether a child's performance was in line with expectations for its age. Those who had spent less time working in child health found it harder to decide whether a child needed a referral to specialist care. Nurses explained that increased experience helped them grasp the broader picture, such that they no longer needed to follow the protocol in detail, and also made them feel more confident in administering the screening.

“easier with the years, I think I was stricter and more finicky at first/… but as long as the child has a name for it, I think it's OK, or if you can sort of hear that language has got going and there's a desire to talk and to make yourself understood to your family in some way.” (Informant 14)

#### 4.1.2 A need for complementary approaches to capture the overall picture

Adding other approaches to capture the child's overall language and communicative ability was described as important by the nurses. They had sometimes felt that the screening was not enough on its own to assess a child's ability to communicate and interact with others. Complementary approaches mentioned included contacting the child's pre-school teacher for information, visiting the child's pre-school to observe its interaction with peers and observing the parent–child interaction during the health visit. Some nurses had also asked parents to make video-recordings of their child playing at home for the nurse to use in assessing whether the child needed further assessment by other professionals. In addition, nurses used different approaches when a child had difficulties participating during a health visit.

“you can't be too rigid, you must consider the whole picture and listen to what happens in the waiting-room and what the parent describes/… and perhaps the parents have a film to show of the child talking and communicating at home.” (Informant 8)

#### 4.1.3 A family-centred approach gives the best results

The nurses highlighted the importance of making the child and its family feel comfortable and relaxed during the visit in order to ensure that the child will perform to the best of its ability. Engaging the child in a joint activity was useful, as was offering an open box of toys that the child could explore at its own pace. Where a child was unwilling to participate, the nurses avoided forcing it, given the importance of children forming a positive impression of healthcare for the future.

“then you have to change the time of the appointment and make adjustments. I think it can be crucial that they're not tired or hungry.” (Informant 12)

### 4.2 External factors influence administration as well as the assessment and analysis of screening results

The nurses identified various external factors influencing both administration and their assessment and analysis of language-screening results, including the children's abilities, general development and adaptability, their multilingualism (in cases where they spoke a language not known to the nurse) and their parents' thoughts and expectations. Non-personal external factors such as waiting times in healthcare were also mentioned.

#### 4.2.1 The child's abilities affect administration

Children aged 2.5 years can be very different – some are shy and unwilling to speak, others are highly interactive, and some are very determined – meaning that health visits may vary greatly in character and that assessing the children may present nurses with various challenges. Indeed, several nurses considered the health visit at 30 months to be the most challenging of all because of the vast differences between individuals. It is easy to assess a child who is focused, but otherwise a great deal of patience and flexibility is needed. When a child is shy, reserved or has difficulties participating in the assessment, the results of the screening may be uncertain, as they might not accurately reflect the child's actual abilities.

“[the language screening is difficult to perform] if a child is very, very active, and you, the child isn't interested in what I'm showing it but is focusing on something completely different.” (Informant 2)

#### 4.2.2 Language barriers affect administration and assessment

Where the child and its family speak a language not known to the nurse, the parents play a more important role in the screening. Sometimes the nurse needs to communicate with the child through the parent or an interpreter, who translates or conveys what the child is saying. Some nurses noted that working with an interpreter can be difficult because interpreters sometimes translate more than just the task, making it difficult for the nurse to evaluate the content of an answer. Assessment and analysis are more challenging when a child answers in a foreign language, but body language can be useful. The analysis can be more challenging because of uncertainties and difficulties. A further important aspect affecting the assessment of multilingual children is whether the child attends pre-school or not.

To form a comprehensive picture, the nurses may need information from perspectives other than that of the parents, but for a child not attending pre-school there are few potential sources of such information.

“but it's hard for me, isn't it, because the child doesn't speak Swedish but answers in its own language, and then the parents may say that he or she is saying so and so, but then that doesn't quite match what people at the pre-school say.” (Informant 17)

#### 4.2.3 Parents’ perceptions have an impact

Parents' experiences and perceptions influence not only the health visit but also the opportunities to provide support and take action afterwards. Some parents stepped in and took over, answering instead of their child, whereas some had too high expectations of their child's performance. In fact, some parents reprimanded their children, which is unfortunate since it needlessly makes them mindful of their performance.

“to a large extent it's the parents’ attitude to their child in terms of what they should know and not know” (Informant 3)

Some parents preferred to attend a health visit without an interpreter present although they might well have needed one. This made visits more difficult and sometimes caused nurses to feel concern about potential misunderstandings. Further, the nurses identified parents with cognitive difficulties as a group where language screening can be a challenging task, particularly explaining the results to the parents when there is a need for further referral after the screening.

“and parents with cognitive difficulties, that's also very hard for us, because they're usually much more suspicious.” (Informant 12)

#### 4.2.4 Decisions are influenced by waiting times in healthcare

Some nurses reported that their decisions regarding referral after a child had failed the screening were influenced by external factors such as waiting times in healthcare. In uncertain cases, they preferred to make a referral to a speech-language pathologist (SLP) rather than taking the risk of missing a child who might need specialist care. They also often told parents that a referral was the best option in uncertain cases, given the long waiting times: by the time the specialist unit responded, the parents would be able to cancel the appointment if they no longer felt their child had any difficulties.

“but it takes so incredibly long before you get an appointment,/… so, well, we might just as well send it off now because it takes such a long time, and in case things sort themselves out then that's great, then you can cancel the appointment.” (Informant 1)

## 5 Discussion

The present study aimed to capture child-health nurses' perspectives on their use of language screening. The analysis yielded two main categories: *Experience and flexibility facilitate use of the screening method* and *External factors influence administration as well as the assessment and analysis of screening results*.

It is clear from the first main category that the nurses considered the language screening to be a well-functioning tool for assessing the language development of children at 30 months of age. Those who had worked longer in child health felt more confident using the screening method, but those with less work experience commonly reported complying more carefully with the protocol. These somewhat contradictory findings suggest that, although many nurses take a positive view of the screening, complying with the protocol can be difficult and longer experience seems to be associated with a risk of more often diverging from it. Our findings are in line with those of previous studies (15, 18), that described difficulties in following, or outright failures to follow, the protocols for various language-screening methods in child health. Moreover, assessing fidelity in language screening has also proved difficult for other types of screening methods (18). Failure to follow a screening protocol obviously entails a risk to validity.

The nurses characterised the health visit for 30-month-old children as the most challenging one within the national child-health programme, mostly because many children of this age are reserved, shy and reluctant to interact with the nurse. The methods that nurses may use to put children at ease during health visits were highlighted, with frequent descriptions of the flexible approaches that child-health nurses must take to be able to administer language screenings. The challenges described in our study have also been identified by others (19, 20), and both targeted and flexible strategies for guiding children during health visits have been characterised as successful (19).

Many nurses in the present study stressed the importance of co-operating with a child's pre-school to collect information about the child's speech and language development in order to make better decisions about whether to refer the child to an SLP after a screening. Several noted that assessing the language development of children who did not attend pre-school was more challenging. It is clear from this that the language screening is sometimes hard to use as a stand-alone instrument at the age in question and that child-health nurses must then gather more information from others in order to be able to assess a child's language development. Further, it is obviously important that nurses have adequate “child skills” and can use different strategies to engage children during health visits. The nurses need to apply those skills and strategies in line with their tasks under the Swedish national child-health programme: promoting child health and identifying children in need of targeted interventions for their speech and language development. The complex and challenging nature of child-health nurses' work has been highlighted previously (19, 20), and the use of structured methods such as language screening may facilitate the identification of children with suspected language disorders. However, although the language screening in question involves a manual-based procedure and could therefore be considered structured, there seem to be various challenges in using it and in analysing the results.

Several such challenges or obstacles were highlighted within the main category *External factors influence administration as well as the assessment and analysis of screening results*, particularly regarding children who had difficulties participating during the health visit and regarding children that speak a different language than the nurse. Child-health nurses have an important role in supporting immigrant families (21, 22), who typically speak a different language than the nurse. This often entails a need to communicate with the child through an interpreter or its parents. Such communication was explicitly highlighted by the nurses in our study as making the assessment uncertain and difficult, as also shown in a previous study (23). Further, there are previous reports of healthcare professionals experiencing challenges in communicating with families who do not speak the majority language and feeling frustration when they cannot provide the family-centred care they aim for (24). In a context where most children use a different language than the nurse, it may be challenging in several ways to use a language screening designed for children that only use Swedish as their main language, and it has been suggested that the CHS should screen all languages to which a child is exposed (25). However, although the current method is not specifically designed for children exposed to multiple languages in their immediate environment, the primary focus of the screening is on whether the child can understand simple instructions and whether grammatical development has begun in any language. These important early milestones can be assessed in relation to any language; if necessary, the nurse may have to ask the parents. Still, a majority of the nurses in the present study highlighted the difficulties of using the method concerned with immigrant families. This suggests that there may be a lack of guidance on how to use the method in such cases, and providing child-health nurses with further training on language development and on the method itself could improve its usability. The suggestion to screen all of a child's languages needs to be evaluated further, but given the challenges associated with the use of interpreters that have been identified in the present and many other studies, that suggestion does not seem feasible today.

Globally, children with language disorders are frequently underserved (26). In Sweden, young children's access to SLPs is notoriously limited, with access to essential interventions delayed by waiting times often exceeding one year after referral (8). The nurses in the present study felt that their decisions were influenced by the long waiting times. For instance, they adopted a strategy of “referring rather than waiting” which will artificially inflate queues even further. That children who may not really need to see an SLP are referred anyway is a troublesome yet understandable finding. At a more general level, the use of screening can be called into question when timely interventions afterwards cannot be guaranteed (27).

Some limitations of the present study need to be pointed out. All participants were from only two geographical areas, which might reduce transferability. However, those areas were purposely chosen to capture experiences from outside the main city of the region. In future research, a survey-based study could be a way to reach a larger part of the region, both urban and rural areas. Additionally, the use of online interviews could be seen as a limitation, since it can be challenging to capture in-depth experiences online. However, the interview material was considered rich and nuanced, suggesting that the online method may not have had a negative impact.

Dependability was enhanced by the use of a question guide for all interviews, ensuring that the study objectives remained aligned and focused. Quotations from the interviews were included in the results section, strengthening confirmability and credibility by demonstrating a clear connection between the interview data and the results (28).

## 6 Conclusion

The present study has raised questions about whether early language screening detects the children it is intended to identify, an issue also discussed in previous studies (7, 15). While the purpose of the present study was not to investigate the validity of the screening, one important finding from it is that many nurses described problems using the screening with children who have difficulties participating during the visit or interacting with the nurse or who have a different language than the nurse. This finding could inform future studies on how early language should be assessed within the CHS.

Furthermore, the finding that experienced nurses found the screening easy to administer, yet were more likely to deviate from the protocol, is noteworthy and should be taken into account when designing clinical professional training on the method.

Early language screening is implemented across all regions in Sweden as part of the national child-health programme, aiming to ensure timely and equitable identification of children in need of support. Our findings show that nurses' experiences with one of the two main methods for such screening differ depending on their professional background and the children's abilities. Immigrant children pose particular challenges, especially those not attending pre-school. Further, nurses reported that their decisions on referral after a failed screening were influenced by external factors, including healthcare waiting times and the time they had spent working in child health. To ensure equal healthcare, the approaches and methods for language screening need to be further developed, especially so as to meet the needs of all children and families residing in today's society.

## Data Availability

The raw data supporting the conclusions of this article will be made available by the authors, without undue reservation.
